# VEGF Stimulates RCAN1.4 Expression in Endothelial Cells via a Pathway Requiring Ca^2+^/Calcineurin and Protein Kinase C-δ

**DOI:** 10.1371/journal.pone.0011435

**Published:** 2010-07-06

**Authors:** Katherine Holmes, Elinor Chapman, Violaine See, Michael J. Cross

**Affiliations:** 1 Department of Pharmacology and Therapeutics, School of Biomedical Sciences, University of Liverpool, Liverpool, United Kingdom; 2 North West Cancer Research Institute, School of Biological Sciences, Bangor University, Bangor, United Kingdom; 3 School of Biological Sciences, University of Liverpool, Liverpool, United Kingdom; Universität Würzburg, Germany

## Abstract

**Background:**

Vascular endothelial growth factor (VEGF) has previously been shown to upregulate the expression of the endogenous calcineurin inhibitor, regulator of calcineurin 1, variant 4 (RCAN1.4). The aim of this study was to determine the role and regulation of VEGF-mediated RCAN1.4 expression, using human dermal microvascular endothelial cells (HDMECs) as a model system.

**Methodology/Principal Findings:**

We show that VEGF is able to induce RCAN1.4 expression during cellular proliferation and differentiation, and that VEGF-mediated expression of RCAN1.4 was inhibited by the use of inhibitors to protein kinase C (PKC) and calcineurin. Further analysis revealed that siRNA silencing of PKC-delta expression partially inhibited VEGF-stimulated RCAN1.4 expression. Knockdown of RCAN1.4 with siRNA resulted in a decrease in cellular migration and disrupted tubular morphogenesis when HDMECs were either stimulated with VEGF in a collagen gel or in an endothelial/fibroblast co-culture model of angiogenesis. Analysis of intracellular signalling revealed that siRNA mediated silencing of RCAN1.4 resulted in increased expression of specific nuclear factor of activated T-cells (NFAT) regulated genes.

**Conclusions/Significance:**

Our data suggests that RCAN1.4 expression is induced by VEGFR-2 activation in a Ca^2+^ and PKC-delta dependent manner and that RCAN1.4 acts to regulate calcineurin activity and gene expression facilitating endothelial cell migration and tubular morphogenesis.

## Introduction

Angiogenesis is defined as the formation of new blood vessels from pre-existing vessels, and is an essential process in embryonic development and normal physiology. However, an unbalance in angiogenesis may play a role in a number of pathological conditions, including cancer, atherosclerosis, and ischaemia [1]. The vascular endothelial growth factor (VEGF) family of homodimeric glycoproteins have been shown to be critical for angiogensis. The VEGF family comprises 5 members; VEGF-A, VEGF-B, VEGF-C, VEGF-D and placenta growth factor (PLGF). In addition there are a number of structurally related proteins, including parapoxvirus *Orf* VEGF (VEGF-E). These ligands bind in an overlapping pattern to 3 receptors; VEGF Receptor 1 (VEGFR-1), VEGFR-2 and VEGFR-3 (reviewed in [2]). Vascular endothelial cells express both VEGFR-1 and VEGFR-2, with VEGFR-2 generally accepted as the principal receptor through which VEGF signals are transmitted in the vascular endothelium. Binding of VEGF to VEGFR-2 results in the activation of a number of intracellular signalling pathways including mitogen-activated protein kinases (MAPKs) and protein kinase B (PKB)/Akt. VEGFR-2 also activates phospholipase C-γ (PLCγ) leading to an increase in intracellular calcium and activation of protein kinase C (PKC; reviewed in [2]).

Proteins within the regulator of calcineurin (RCAN) family are able to bind and regulate the protein phosphatase calcineurin. This family comprises 3 members; RCAN1, 2 and 3. RCAN1 was initially given the name Down syndrome critical region 1 (DSCR1) due to its location on chromosome 21 [3]. Other names include Adapt 78, myocyte-enriched calcineurin interacting protein 1 (MCIP1) and calcipressin 1 [4], [5], [6].

The human *RCAN1* gene comprises 7 exons, the first 4 of which are alternative first exons, resulting in different isoforms which show different patterns of expression and regulation [7]. Exon one gives rise to the isoform RCAN1.1 [8]. Exon 2 lacks a methionine start site required for translation, and exon 3 encodes just 3 amino acids [7]. Exon 4 gives rise to the isoform RCAN1.4, and is under the control of a calcineurin responsive promoter, comprising multiple consensus binding sites for the transcription factor nuclear factor of activated T-cells (NFAT) [9] and GATA-2/3 sites [10]. More recently, 5 consensus binding sites for activator protein 1 (AP-1) transcription factors have been identified in the region flanking exon 4 [11].

The serine/threonine protein phosphatase 2B (PP2B)/calcineurin is a heterodimer composed of a calcineurin catalytic subunit A (CnA), and a calcineurin regulatory subunit B (CnB). Upon Ca^2+^ induced activation, CnA dephosphorylates members of the NFAT family [12]. This dephosphorylation allows the translocation of NFAT to the nucleus where it binds to the NFAT consensus sequence present in the promoter region of various genes, including RCAN1.4, resulting in an increase in transcription [12]. RCAN1.4 binds to the catalytic domain within CnA and inhibits its activity [5], [6], [13], [14]. Phosphorylation of RCAN1.4 by MAPK and glycogen synthase kinase 3 (GSK-3), allows RCAN1.4 to act as a substrate for CnA [15]. Thus, RCAN1.4 has been suggested to act as a negative feedback inhibitor for CnA signalling.

RCAN1 has previously been shown to be upregulated in a variety of endothelial cell lines in response to VEGF, including human umbilical vein endothelial cells (HUVEC), human aortic endothelial cells (HAEC), human dermal microvascular endothelial cells (HDMEC), and human retinal endothelial cells (HREC) [10], [16], [17]. In each of these studies RCAN1.4, but not RCAN1.1 was found to be upregulated in response to VEGF treatment. Knockdown of RCAN1 in endothelial cells has also been shown to inhibit VEGF stimulated migration *in vitro*, and VEGF induced angiogenesis, *in vivo* [16]. Most recently, an increase in *Rcan1* expression in mice, by the introduction of an extra transgenic copy, has been shown to suppress tumour growth [18]. In this study, we have analysed the intracellular signalling pathways utilised by VEGFR-2 to stimulate RCAN1.4 expression in primary human dermal microvascular endothelial cells (HDMECs). Our results show that the calcineurin/NFAT pathway and PKC-delta pathway are required for maximal induction of RCAN1.4. Furthermore, RCAN1.4 was required for migration and efficient tubular morphogenesis in these cells.

## Materials and Methods

### Materials

Recombinant human VEGF-A_165_, epidermal growth factor (EGF), hepatocyte growth factor (HGF) and colony stimulating factor-1 (CSF-1) were obtained from Peprotech. Recombinant human VEGF-B_167_ and recombinant *Orf* virus VEGF-E were obtained from Promokine. Fibroblast growth factor-2 (FGF-2) was obtained from R&D Systems. Phorbol-12-myristate-13-acetate (PMA), A23187, GF109203X (GFX), Gö6976, Cyclosporin A (CsA) and BAPTA-AM were obtained from Calbiochem. ZM323881 was obtained from Tocris. Oleoyl-L-α-lysophosphatidic acid (LPA) and ethylene glycol tetraacetic acid (EGTA) were obtained from Sigma. All other materials were obtained from Invitrogen unless otherwise stated.

### Cell Culture

Human dermal microvascular endothelial cells (HDMEC), human umbilical vein endothelial cells (HUVEC) and human aortic endothelial cells (HAEC) were purchased from Promocell and were cultured in endothelial cell basal media (EBM) MV2 growth media (C-22221; Promocell), supplemented with 5% (v/v) foetal calf serum (FCS) and EGF (5 ng/ml), VEGF (0.5 ng/ml), FGF-2 (10 ng/ml), long R3 insulin growth factor-1 (20 ng/ml), hydrocortisone (0.2 µg/ml) and ascorbic acid (1 µg/ml) (supplement pack C-39221; Promocell). HEK 293 cells were cultured in Dulbecco's' Modified Eagles Medium (DMEM) supplemented with 10% FCS. Human dermal fibroblasts were obtained from Promocell, and cultured in fibroblast growth media (C-23010; Promocell) supplemented with FGF-2 (1 ng/ml) and Insulin (5 µg/ml) (supplement pack; Promocell). Cells were routinely cultured on gelatin coated plates in a humidified incubator under 5% CO_2_ at 37°C. For growth factor stimulation, cells were serum starved for at least 16 hours by changing media to MV2 media supplemented with 1% (v/v) FCS without growth factor supplements.

### Quantitative Real Time-Polymerase Chain Reaction (RT-qPCR)

Total RNA was extracted from HDMECs using the RNeasy mini kit (Qiagen). DNase treatment was performed using the on-column DNase digestion (Qiagen). One µg total RNA was used for cDNA synthesis with M-MLV reverse transcriptase and oligodT primers. RT-qPCR was performed using Power SYBR Green Mastermix (Applied Biosystems) and the primer sequences as detailed below. Reactions were analysed on an ABI 7000 real-time PCR machine using the following cycle conditions; 2 minutes at 50°C, 10 minutes at 95°C, followed by 45 cycles of 15 seconds at 95°C and 60 seconds at 60°C. Results were normalised against *β-actin* mRNA expression. Primer sequences were as follows; RCAN1.4 forward –CTCACTAGGGGCTTGACTGC, reverse - CAGGCAATCAGGGAGCTAAA; RCAN1.1 forward – TCATTGACTGCGAGATGGAG, reverse – TGATGTCCTTGTCATACGTCCT; COX-2 forward – GAAGAAAGTTCATCCCTGATCCC, reverse – CTGGGCAAAGAATGCAAACA; IL-8 forward – CAGGAATTGAATGGGTTTGC, reverse – AGCAGACTAGGGTTGCCAG; ICAM-1 forward – CAAGGCCTCAGTCAGTGTGA, reverse – CCTCTGGCTTCGTCAGAATC.

### Immunoblotting

Protein lysates from HDMECs were prepared in LDS sample buffer containing β-mercaptoethanol. Proteins were resolved by SDS-PAGE on 4–12% NuPage Gels, and transferred onto nitrocellulose membranes (Amersham). Membranes were blocked with 5% (w/v) BSA. Proteins were detected using the following antibodies; RCAN1 (D6694; Sigma), NFAT1 (610702; BD Transductions Labs), phospho-p44/42 MAPK (Thr202/Tyr204; #4377), phospho-PKC (pan; #9371), phospho-PLCγ (Tyr 783; #2821), PKCα (#2056), PKCδ (∼2058; all Cell Signalling Technology), PKCε (06-991; Upstate), PKCη (sc-215), colony stimulating factor-1 receptor (CSF-1R; sc-13949) and Actin (sc-1625; all Santa Cruz Biotechnology). Membranes were washed 6 times with TBS-T, and incubated with peroxidise-conjugated secondary antibodies (Amersham/Sigma). Blots were detected using an enhanced chemiluminescence (ECL) detection kit (Amersham).

### siRNA transfection

HDMECs were transfected with either two separate small interfering RNA (siRNA) duplexes at a concentration of 10 nM, or a mixture of two siRNA duplexes at a concentration of 10 nM each, using 0.1% (v/v) Lipofectamine RNAiMAX, according to the manufacturer's instructions. All siRNA duplexes were validated by RT-qPCR and western blotting prior to use [19]. Transfection reactions were performed in serum-free OptiMEM. Cell media was changed to serum containing media 4 hours after transfection. The following siRNA duplexes were obtained from Qiagen; RCAN1 (Hs_DSCR1_5 HP, SI03224900; Hs_DSCR1_6 HP, SI03246208), PKCα (Hs_PRKCA_5 HP, SI00301308; Hs_PRKCA_6, HP SI00605927), PKCδ (Hs_PRKCD_8 HP, SI00288337; Hs_PRKCD_11 HP, SI02660539), PKCε (Hs_PRKCE_5 HP, SI00287784; Hs_PRKCE_6 HP, SI02622088), and PKCη (Hs_PRKCH_5, SI02224075; Hs_PRKCH_6 HP, SI02224082) and All-stars Negative Control siRNA.

### Overexpression of Chimeric Receptors

The pcDNA3.1 mammalian expression plasmids encoding the extracellular domain of human CSF-1 receptor (CSF-1R)/c-fms fused to the transmembrane and intracellular domain of murine VEGFR-2 (CKRwt) and murine VEGFR-2 Y1173F point mutation (CKR Y1173F mutant) were kindly provided by Dr. Nader Rahimi (Boston University, Boston, MA). HDMEC were combined with the plasmid DNA and HMVEC-L Nucleofector™ solution (AMAXA) according to the manufacturer's instructions and transfected using program S-05 on the AMAXA Nucleofector™. Cells were then transferred to cell culture dishes and allowed to adhere in 1% FCS containing media overnight.

### Intracellular Calcium Measurements

HDMECs plated upon glass based cell culture dishes (Iwaki) were loaded with the Ca^2+^ indicator dyes Fluo-4-AM (5 µM) and Fura Red-AM (5 µM) for 20 minutes. Intracellular calcium was monitored using fluorescent microscopy to measure the emission ratio of Fluo-4/Fura Red.

### Inositol Phosphate assay

PLC activity was determined by measuring the agonist-stimulated accumulation of [^3^H]inositol phosphates in the presence of 20 mM LiCl from [^3^H]*myo*-inositol labelled cells as previously described [20].

### Cellular Proliferation Assay

HDMECs were seeded in 24 well plates in normal growth media for 24 hours. Media was then changed to 1% (v/v) FCS containing media for 24 hours. Cells were stimulated with VEGF (50 ng/ml) for 72 hours, and the number of viable cells determined using a luminescent based Cell-titre glo assay (Promega).

### Scratch Wound Migration Assay

HDMECs were seeded in 12 well plates in normal growth media for 48 hours. Media was then changed to 1% (v/v) FCS containing media for 24 hours. A scratch was introduced to the cell monolayer using a sterile 200 µl pipette tip. Cells were stimulated with VEGF (50 ng/ml) for 18 hours. The cells were then fixed with 2% (w/v) paraformaldehyde and then stained with 0.5% (w/v) crystal violet solution to allow enhanced imaging.

### Tube Formation Assay

Collagen gels were prepared by mixing bovine collagen type-I (Vitrogen) with 0.1 M NaOH and 10x Ham's F-12 media (Promocell) in the ratio 8∶1∶1, and supplemented with 20 mM HEPES, 0.117% (w/v) bicarbonate solution, and 2 mM Glutamax-I. HDMEC were starved in 1% (v/v) FCS containing media for 24 hours prior to being seeded onto pre-prepared collagen gels. Once cells had adhered, a top layer of collagen was added and allowed to set. Cells were then stimulated with growth factors in 1% (v/v) FCS containing media for 20 hours to induce tubular morphogenesis. Collagen gels were fixed with 2% (w/v) paraformaldehyde and cells visualized by phase contrast microscopy.

### Co-culture Assay

Human dermal fibroblasts were seeded in 24 well plates, and allowed to become confluent. On day 4 HDMEC were seeded upon the fibroblast monolayer. Co-cultures were serum starved, and then stimulated with VEGF for 5 days. To assess the effects of gene knockdown siRNA transfections were performed upon days 3, 5 and 8. In order to visualise tube formation by HDMEC co-cultures were fixed with 70% (v/v) ethanol, and then stained with the endothelial specific marker CD31 (Dako) [21].

### Statistics

Results are expressed as mean ± standard deviation (S.D.). Statistical analyses were performed using an unpaired Student's *t-*test. Statistical significance was set at a P-value less than 0.05 (*), or 0.01 (**).

## Results

### VEGF-A, PMA and A23187 stimulate an increase in RCAN1.4 expression in HDMEC

**Figure 1:**
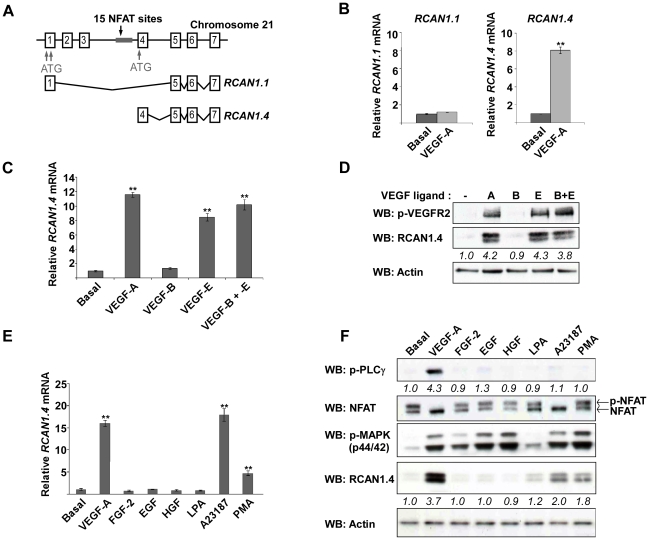
VEGF-A stimulates RCAN1.4 expression, PLC-γ activation and NFAT dephosphorylation in HDMEC. (*A*) Exon structure of RCAN1, showing the alternative first exons used by the RCAN1.1 and RCAN1.4 isoforms. (*B*) HDMEC were stimulated with VEGF-A (50 ng/ml) for 1 hour, and the increase in expression of *RCAN1.1* and *RCAN1.4* mRNA, relative to the level of each mRNA in unstimulated (basal) cells, determined by RT-qPCR. Results were normalised to *β-actin* mRNA expression. Data shows mean ± SD (n = 3). **P<0.01, versus Basal. (*C*) HDMEC were stimulated with VEGF-A (50 ng/ml), VEGF-B (50 ng/ml), VEGF-E (50 ng/ml) or VEGF-B and –E (50 ng/ml each) for 1 hour and the expression of *RCAN1.4* mRNA determined by RT-qPCR. Results were normalised to *β-actin* mRNA expression. Data shows mean ± SD (n = 3). **P<0.01, versus Basal. (*D*) HDMEC were stimulated with agonists as in 1*C* for 10 mins or 3 hours and the expression levels of RCAN1.4 (3 hour stimulation), and phospho-VEGFR2 (pVEGFR2) proteins determined by SDS-PAGE followed by western blotting. Actin was used as a loading control. Numbers below the blots denote the relative density of each band. Results are representative of 3 experiments. (*E*) HDMEC were stimulated with VEGF-A (50 ng/ml), FGF-2 (50 ng/ml), EGF (50 ng/ml), HGF (50 ng/ml), LPA (10 µM), A23187 (5 µM) and PMA (100 nM) for 1 hour, and the expression of *RCAN1.4* mRNA, relative to unstimulated (basal) cells, determined by RT-qPCR. Results were normalised to *β-actin* mRNA expression. Data shows mean ± SD (n = 3). **P<0.01, versus Basal. (*F*) HDMEC were stimulated with agonists as in 1*C* for 10 mins or 3 hours and the expression levels of RCAN1.4 (3 hour stimulation), phospho–PLCγ (p-PLCγ), NFAT and phospho-p44/42 MAPK (p-MAPK) proteins determined by SDS-PAGE followed by western blotting. Actin was used as a loading control. Numbers below the blots denote the relative density of each band. Results are representative of 3 experiments.

Human *RCAN1* comprises seven exons, the first four of which are alternatively spliced to give rise to different isoforms (Fig. 1A). Quantitative RT-PCR was used to analyse the expression of *RCAN1.1* and *RCAN1.4* mRNA in response to VEGF-A in HDMECs. Under basal conditions, the amount of *RCAN1.4* mRNA was found to be 100-fold greater than the amount of *RCAN1.1* mRNA (data not shown). Following VEGF-A stimulation, levels of *RCAN1.4* mRNA were increased 8-fold (Fig. 1B); *RCAN1.1* mRNA levels were not increased by VEGF-A stimulation (Fig. 1B). The expression of RCAN1.1 protein, which was practically undetectable on a western blot using an antibody to the C-terminal of RCAN1 which is able to detect all RCAN1 isoforms, did not increase upon VEGF-A stimulation (data not shown).

Vascular endothelial cells express both VEGFR-1, and VEGFR-2, both of which are receptors for VEGF-A. Expression of both receptors in HDMEC was confirmed by PCR (data not shown). In order to establish the role of each receptor in RCAN1.4 upregulation, VEGF-B and VEGF-E, which bind VEGFR-1 and VEGFR-2 respectively, were used to stimulate HDMEC. VEGF-B did not elicit an RCAN1.4 upregulation, while VEGF-E was able to induce a similar increase in RCAN1.4 expression as VEGF-A at both the mRNA and protein level (Fig. 1C and D). For the purposes of this study, all further experiments were conducted using VEGF-A.

In contrast to VEGF-A, stimulation of HDMECs with a range of growth factors (FGF-2, EGF and HGF), and the G-protein agonist LPA did not evoke an increase in *RCAN1.4* mRNA (Fig. 1E) or RCAN1.4 protein (Fig. 1F); active signalling by all growth factors was confirmed by MAPK phosphorylation. VEGF-A was also unique in its ability to stimulate PLC-γ phosphorylation and NFAT activation (Fig. 1F). A23187, a Ca^2+^ ionophore, and PMA, a potent PKC activator, were capable of inducing increased RCAN1.4 mRNA (Fig. 1E) and increased RCAN1.4 protein levels in the absence of increased PLC-γ phosphorylation (Fig. 1F). Interestingly, unlike VEGF-A and A23187, PMA did not appear to stimulate the dephosphorylation of NFAT.

### RCAN1.4 is downstream of PLC-γ, PKC and Ca^2+^/Calcineurin

**Figure 2:**
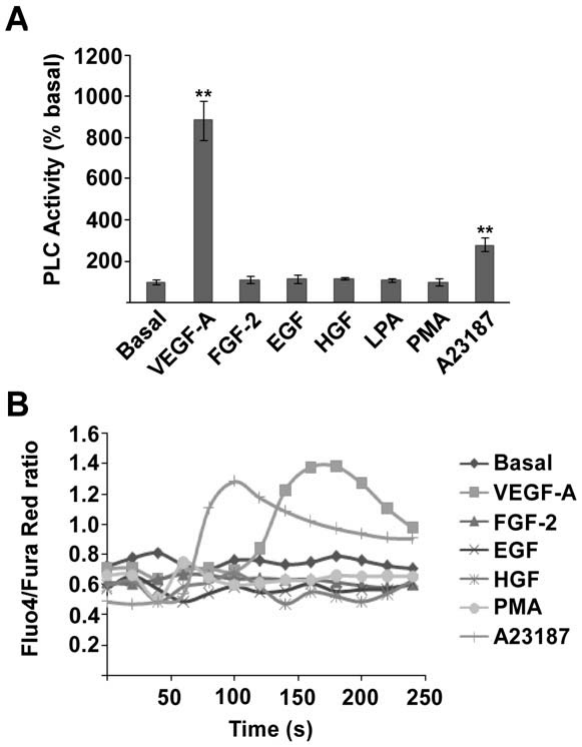
VEGF-A stimulates PLC activity and Ca^2+^ release. (*A*) HDMEC were stimulated with VEGF-A (50 ng/ml), FGF-2 (50 ng/ml), EGF (50 ng/ml), HGF (50 ng/ml), LPA (10 µM), A23187 (5 µM) and PMA (100 nM) for 30 mins, and PLC activity determined by inositol phosphate assay. Data shows mean ± SD (n = 3). **P<0.01, versus Basal. (*B*) HDMEC were loaded with Fluo4 and Fura Red dyes and stimulated with agonists as in 2*A* and changes in intracellular calcium monitored by measuring the fluorescent intensity of the two dyes. Results are representative of 3 experiments.

The ability of VEGF-A to uniquely stimulate PLC activity in HDMECs compared with other growth factors was verified by analysing the generation of inositol phosphates (Fig. 2A). This data correlates with the finding that increases in intracellular Ca^2+^ were only observed following stimulation with VEGF-A and A23187, a Ca^2+^ ionophore (Fig. 2B).

**Figure 3:**
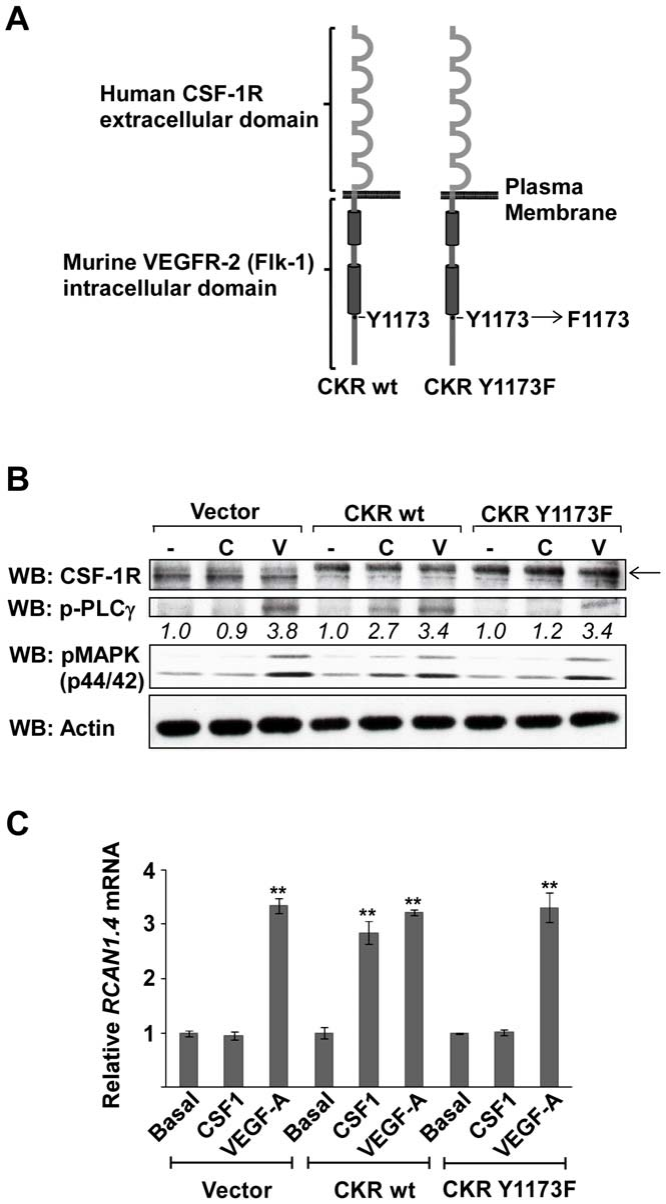
Induction of RCAN1.4 requires phosphorylation of Y1175 on VEGFR-2. (*A*) Structure of chimeric receptors used to transfect HDMEC. (*B*) HDMEC were transfected with chimeric receptors and stimulated with CSF-1 (C; 50 ng/ml) or VEGF-A (V; 50 ng/ml) for 10 mins. The levels of CSF-1R, phospho-PLCγ (p-PLCγ) and phospho-p44/42 MAPK (pMAPK) were determined by SDS-PAGE followed by western blotting. Actin was used as a loading control. Numbers below the blots denote the relative density of each band. Results are representative of 3 experiments. (*C*) HDMEC were transfected with chimeric receptors, stimulated with CSF-1 (50 ng/ml) or VEGF-A (50 ng/ml) for 1 hour, and the expression of *RCAN1.4* mRNA, relative to unstimulated vector transfected cells, determined by RT-qPCR. Results were normalised to *β-actin* mRNA expression. Data shows mean ± SD (n = 3). **P<0.01, versus unstimulated vector control.

Phosphorylation of the tyrosine 1175 site in VEGFR-2, following VEGF-A stimulation, allows the binding and subsequent phosphorylation of PLC-γ [22]. PLC-γ is then able to catalyze the hydrolysis of the membrane phospholipid phosphatidylinositol (4,5)-bisphosphate (PIP_2_) resulting in the generation of diacylglycerol (DAG), and inositol 1,4,5-trisphosphate (IP_3_). DAG is a physiological activator of PKC, whilst IP_3_ acts upon the endoplasmic reticulum to release calcium stores, resulting in a rise in intracellular calcium ([Ca^2+^]_i_). In order to further assess the importance of PLC-γ activation by VEGFR-2 in the increase of RCAN1.4 expression, HDMECs were transiently transfected using AMAXA nucleofection with plasmids encoding chimeric receptors, comprising the extracellular domain of the human CSF-1R fused with the transmembrane and intracellular domain of the murine VEGFR-2 (Flk-1; Fig. 3A) [23], [24]. Two forms of the chimeric receptor were used; the wild-type (CKRwt), and a mutated form in which the tyrosine residue at position 1173 (corresponding to position 1175 in the human receptor) is replaced with a phenylalanine residue (CKR Y1173F). In cells expressing CKRwt, CSF-1 ligand may be used to elicit a VEGF-A-like response, including phosphorylation of PLC-γ following its binding to tyrosine residue 1173. However, in cells expressing CKR Y1173F, stimulation with CSF-1 can no longer elicit PLC-γ phosphorylation. Expression of the chimeric receptors was confirmed by western blotting with an antibody to the extracellular domain of CSF-1R, and active signalling confirmed by the phosphorylation of PLC-γ and MAPK in response to the ligand CSF-1 (Fig. 3B). Phosphorylated PLC-γ was observed in CKRwt transfected cells in response to CSF-1 stimulation. This phosphorylation was lost in cells transfected with the CKR Y1173F mutant confirming that PLC-γ was not activated by the CKR Y1173F receptor.

The importance of PLC-γ activation in the upregulation of *RCAN1.4* mRNA was assessed by RT-qPCR (Fig. 3C). In cells transfected with CKRwt, levels of *RCAN1.4* mRNA were found to be upregulated when stimulated with CSF-1 to a similar magnitude as that seen when stimulating with VEGF. However, in cells transfected with CKR Y1173F, stimulation with CSF-1 did not induce a rise in the levels of *RCAN1.4* mRNA. Stimulating untransfected cells with CSF-1 did not induce an increase in *RCAN1.4* mRNA, consistent with the lack of expression of endogenous CSF-1R in these cells.

**Figure 4:**
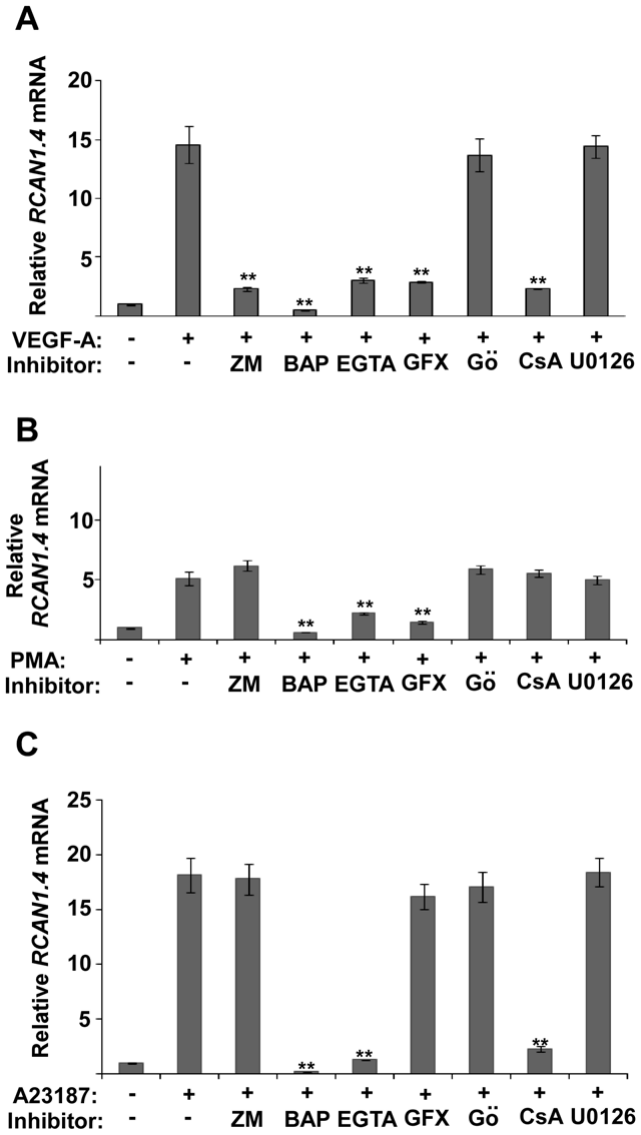
RCAN1.4 induction requires Ca^2+^, calcineurin and PKC activity. HDMEC were incubated with the inhibitors ZM (3 µM), BAP (20 mM), EGTA (2.5 mM), GFX (3 µM), Go (3 µM). CsA (100 nM) or U0126 (5 µM) for 30 minutes, and then stimulated with: (*A*) VEGF-A (50 ng/ml), (*B*) PMA (100 nM), or (*C*) A23187 (5 µM) for 1 hour. Expression of *RCAN1.4* mRNA, relative to unstimulated (basal) cells, was determined by RT-qPCR. Results were normalised to *β-actin* expression. Data shows mean ± SD (n = 3). **P<0.01, versus VEGF/PMA/A23187 treated. BAP – BAPTA/AM, CsA – cyclosporin A, GFX - GF109203X, Go – Gö6976, ZM – ZM323881.

A range of inhibitors were used to further investigate the signalling pathways resulting in the upregulation of RCAN1.4 by VEGF-A (Fig. 4A). Use of ZM323881, an inhibitor to VEGFR-2 [25], inhibited RCAN1.4 upregulation, confirming further that upregulation of RCAN1.4 by VEGF-A was occurring through VEGFR-2 signalling. BAPTA and EGTA, chelators of intracellular and extracellular calcium respectively [26], were both found to inhibit VEGF-A induced RCAN1.4. GF109203X, a broad spectrum PKC inhibitor [27] was found to inhibit VEGF-A induced RCAN1.4, whilst Gö6976, a classical PKC inhibitor [28], did not, implicating a novel PKC in the upregulation of RCAN1.4 expression. Finally cyclosporin A (CsA), a potent inhibitor of calcineurin [29], was found to block VEGF-A induced RCAN1.4. These data support roles for Ca^2+^, calcineurin and PKC in the pathway linking VEGFR-2 to RCAN1.4 expression.

The same inhibitors were used in combination with PMA (Fig. 4B) and A23187 (Fig. 4C). As expected GF109203X inhibited PMA stimulation of RCAN1.4. However, as with VEGF-A, Gö6976 did not affect PMA stimulated RCAN1.4 expression. The use of CsA supported our previous data (Fig. 1D), showing that PMA is not acting through the calcineurin pathway to stimulate expression of RCAN1.4. A23187 induction of RCAN1.4 was inhibited by the Ca^2+^ chelators, and by CsA, but not by PKC inhibitors. Use of the MEK1 inhibitor U0126 [30] did not affect RCAN1.4 induction by VEGF, PMA or A23187, suggesting that RCAN1.4 is not downstream of the MEK-p44/p42 MAPK pathway.

### PKCδ is required for induction of RCAN1.4

**Figure 5:**
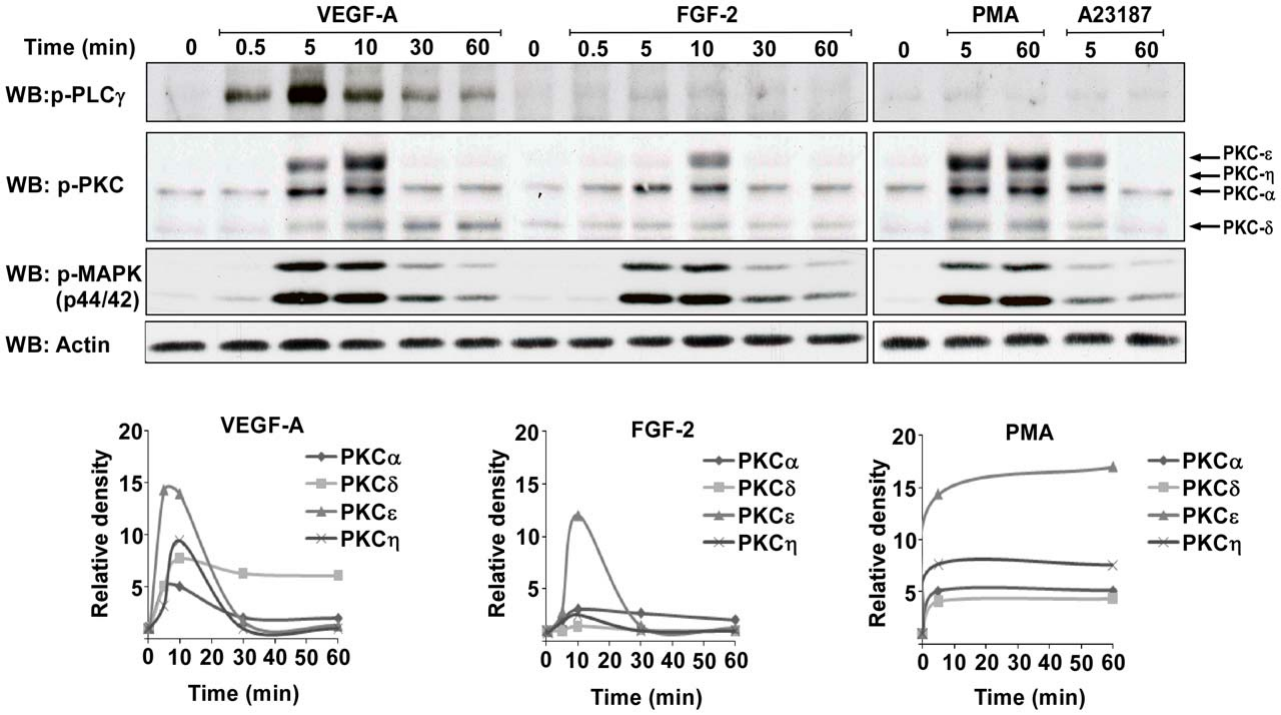
PKCδ phosphorylation is sustained in response to VEGF-A. HDMEC were stimulated with VEGF-A (50 ng/ml), FGF-2 (50 ng/ml) PMA (100 nM) or A23187 (5 µM) for the time points indicated. The expression levels of phospho-PLCγ (p-PLCγ), phospho-PKC (p-PKC) and phospho-p44/42 MAPK (p-MAPK) were determined by SDS-PAGE followed by western blotting. Actin was used as a loading control. Graphs show the relative expression of each phosphorylated PKC isoform in response to either VEGF-A, FGF-2 or PMA, as determined from the relative density of the bands compared to the unstimulated (basal) condition. Results are representative of 3 experiments.

The phosphorylation of different PKC isoforms following stimulation with VEGF-A, FGF-2, PMA or A23187 was investigated using a pan-phospho PKC antibody (Fig. 5). The antibody used detects serine residues within the hydrophobic regions of PKCα, δ, ε and η, the phosphorylation of which is required for PKC activation [31]. PKCα, δ, ε and η were all rapidly phosphorylated in response to VEGF-A. However, only the phosphorylation of PKCδ was found to be sustained above basal levels over a 60 minute period, while the phosphorylation of PKC α, ε and η were found to return to basal levels within 30 minutes (Fig. 5). In response to FGF-2 stimulation, small transient increases in the phosphorylation of PKCα, PKCε and PKCη were observed (Fig. 5). As with VEGF-A, the phosphorylation of these isoforms returned to basal levels within 30 minutes of stimulation. Similar data to FGF-2 was obtained when stimulating HDMECs with EGF (data not shown). In response to PMA, the phosphorylation of all PKC isoforms is sustained over 60 minutes. In contrast, in response to A23187 the PKCs were seen to undergo a weak transient phosphorylation.

**Figure 6:**
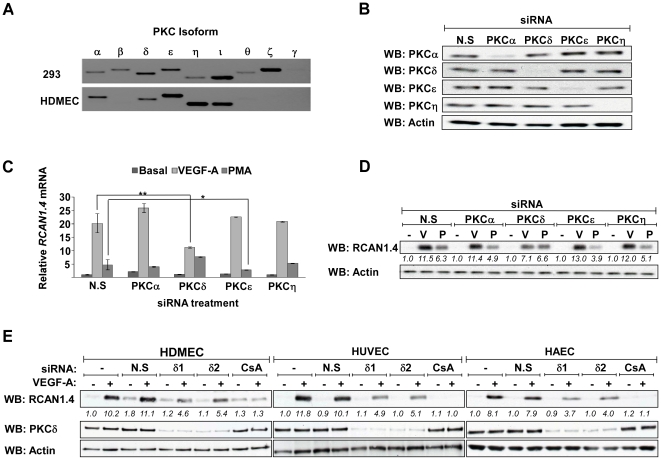
VEGF-A mediated RCAN1.4 expression is regulated by PKCδ. (*A*) PKC isoforms present in HDMEC and 293 cells were quantified by endpoint PCR run on an agarose gel. Results are representative of 3 experiments (*B*) HDMEC were transfected with a mixture of two siRNAs to individual PKC isoforms or with non-silencing (N.S.) siRNA, and the knockdown confirmed by SDS-PAGE followed by western blotting. Actin was used as a loading control. Results are representative of 3 experiments (*C*) HDMEC were transfected with a mixture of two siRNAs to individual PKC isoforms and stimulated 48 hours post-transfection with VEGF-A (50 ng/ml) or PMA (100 nM) for 1 hour. Expression of *RCAN1.4* mRNA, relative to unstimulated (basal) N.S. transfected cells, was determined by RT-qPCR. Results were normalised to *β-actin* expression. Data shows mean ± SD, (n = 3). *P<0.05, **P<0.01, versus N.S. treated cells. (*D*) HDMEC were transfected with a mixture of two siRNAs to individual PKC isoforms and stimulated 48 hours post-transfection with VEGF (V; 50 ng/ml) or PMA (P; 100 nM) for 3 hours. Expression levels of RCAN1.4 protein were determined by SDS-PAGE followed by western blotting. Actin was used as a loading control. Numbers below the blot denote the relative density of each band. Results are representative of 3 experiments. (*E*) HDMEC, HUVEC and HAEC were transfected with two separate siRNAs to PKCδ or N.S siRNA as indicated, and stimulated 48 hours post-transfection with VEGF (50 ng/ml) for 3 hours. Expression levels of RCAN1.4 protein were determined by SDS-PAGE followed by western blotting. Actin was used as a loading control. Numbers below the blots denote the relative density of each band. Results are representative of 3 experiments.

Use of the PKC activator, PMA, and PKC inhibitors, GF109203X and Gö6976, implicated the involvement of a novel PKC in the upregulation of RCAN1.4. PKC is a multi-protein family, comprising several isoforms [32]. The classical isoforms α, β, and γ have a requirement for Ca^2+^ and DAG for activity, whereas the novel isoforms δ, ε, η and θ require DAG but not Ca^2+^. A third class of atypical isoforms require neither Ca^2+^ nor DAG. The PKC expression profile in HDMECs was characterised using end-point PCR, and visualised on an agarose gel (Fig. 6A). The PKC profile of 293 cells was also characterised as a positive control for the primer sets used. HDMECs were found to express the classical PKC isoform α, the novel isoforms δ, ε, and η, and the atypical isoform ι.

HDMEC were transfected with siRNAs to the expressed classical (α) and novel isoforms (δ, ε and η) using non-silencing (N.S.) siRNA as a control. The knockdown of each isoform was confirmed by western blotting (Fig. 6B). Knockdown of each isoform was quantified by RT-qPCR, and found to be between 80 and 90% (data not shown). Knockdown of PKCδ was found to reduce VEGF-A upregulation of RCAN1.4 mRNA by approximately 50% (Fig. 6C). Knockdown of other PKC isoforms present in HDMECs did not affect VEGF-A upregulation of RCAN1.4. This was confirmed at the protein level by western blotting, with a 40% reduction in RCAN1.4 protein observed following PKCδ knockdown (Fig. 6D). Knockdown of PKCδ was not found to decrease the ability of PMA to stimulate RCAN1.4. However, knockdown of PKCε reduced PMA stimulated RCAN1.4 mRNA (Fig. 6C), and reduced protein levels by 40% (Fig. 6D).

In order to confirm that the role of PKCδ in VEGF-A stimulated upregulation of RCAN1.4 is not confined to HDMEC, PKCδ knockdown was also performed in HUVEC and HAEC using two separate PKCδ specific siRNA duplexes (Fig. 6E). In all 3 endothelial cell lines knockdown of PKCδ resulted in an approximate 50% reduction in RCAN1.4 expression in response to VEGF-A. In addition, CsA was successfully used to inhibit RCAN1.4 upregulation in all 3 cell lines (Fig. 6E).

### Knockdown of RCAN1.4 affects expression of NFAT regulated genes

**Figure 7:**
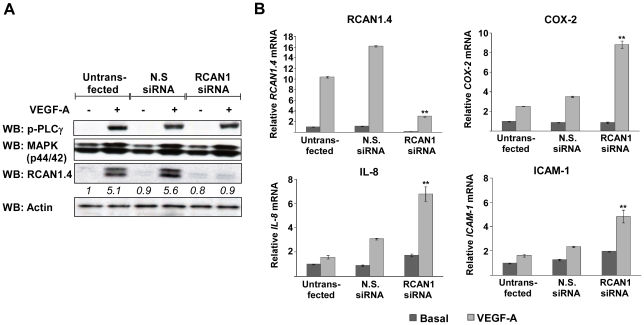
RCAN1.4 regulates NFAT induced gene expression. (*A*) HDMEC were transfected with a mixture of two siRNAs to RCAN1 or non-silencing (N.S.) siRNA and the knockdown of RCAN1.4 and effects on phospho-PLCγ (p-PLCγ) and phospho-p44/42 MAPK (pMAPK) were determined by SDS-PAGE followed by western blotting. Actin was used as a loading control. Numbers below the blots denote the relative density of each band. Results are representative of 3 experiments. (*B*) HDMEC were transfected with a mixture of two RCAN1 siRNAs and stimulated with VEGF-A (50 ng/ml) for 1 hour. Expression of *COX-2*, *IL-8* and *ICAM-1* mRNA, relative to unstimulated (basal) untransfected cells, were determined by RT-qPCR. Results were normalised to *β-actin* mRNA expression. Data shows mean ± SD (n = 3). **P<0.01, versus untransfected.

RCAN1.4 has previously been shown to regulate calcineurin signalling via a negative feedback loop [5], [6], [9]. In order to investigate the role of RCAN1.4 in the regulation of VEGFR-2 signalling we utilised siRNA against RCAN1 and analysed signalling and gene expression in response to VEGF-A. Due to the lack of availability of isoform specific RCAN1.4 siRNAs, the two duplexes used in this study are capable of knocking down the expression of both RCAN1 isoforms. Since RCAN1.4 is the only detectable isoform and is VEGF-inducible, we attribute the effects of the siRNA on VEGF signalling to changes in RCAN1.4 expression. Efficient knockdown of RCAN1.4 was confirmed at the protein level by western blotting (Fig. 7A) and at the mRNA level by qRT-PCR with a 90% reduction in mRNA compared with the N.S. siRNA control (data not shown). No effect of RCAN1.4 knockdown was observed on the phosphorylation of PLC-γ and MAPK in response to VEGF (Fig. 7A). In order to analyse the effect of RCAN1.4 silencing on gene expression we chose a panel of NFAT-regulated genes: *COX-2* [33], *IL-8* [34] and *ICAM-1* [35]. RCAN1.4 knockdown increased the expression of *COX-2*, *IL-8* and *ICAM-1* mRNA in response to VEGF-A (Fig. 7B), confirming that RCAN1.4 acts as a negative regulator of NFAT-mediated gene expression.

### Knockdown of RCAN1.4 affects cellular migration and tubular morphogenesis, but not cellular proliferation

**Figure 8:**
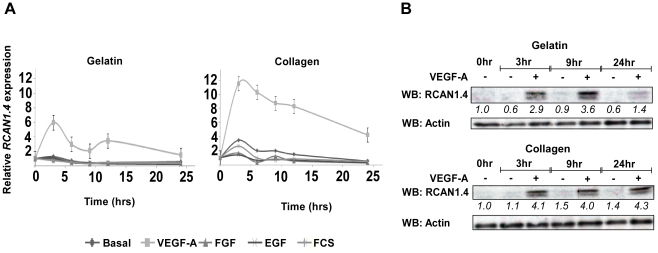
RCAN1.4 is expressed during VEGF-mediated proliferation and tubular morphogenesis. (*A*) HDMEC were plated onto either a collagen or gelatin matrix and stimulated with VEGF-A (50 ng/ml), FGF-2 (50 ng/ml), EGF (50 ng/ml) or FCS (10%), for the time points indicated. Expression of *RCAN1.4* mRNA, relative to unstimulated (basal) cells at 0 hr, was determined by RT-qPCR. Results were normalised to *β-actin* mRNA expression. Data shows mean ±SD, (n = 3). (*B*) HDMEC were treated as in 8(*A*), and the expression of RCAN1.4 protein determined by SDS-PAGE followed by western blotting. Numbers below the blots denote the relative density of each band. Results are representative of 3 experiments.

It has previously been shown that plating telomerase-immortalised HDMEC onto either a collagen or fibronectin matrix causes the cells to respond differently [36]. Upon a collagen matrix the cells undergo tubular morphogenesis in response to VEGF-A stimulation, whereas on a fibronectin matrix the cells proliferate in response to VEGF-A. In our studies, we have used gelatin, rather than fibronectin, as a similar proliferative behaviour was exhibited upon each matrix. The expression of *RCAN1.4* mRNA in response to VEGF-A upon collagen and gelatin matrices over a 24 hour period was determined using RT-qPCR (Fig. 8A). *RCAN1.4* mRNA expression was increased upon both matrices. However, the increase in expression was greater on the collagen matrix, as opposed to the gelatin matrix. In addition, the expression of *RCAN1.4* mRNA was sustained for 24 hours on collagen, whereas on the gelatin matrix expression levels had returned to close to basal levels within 24 hours. A similar pattern of RCAN1.4 expression was observed at the protein level on both matrices (Fig. 8B).

**Figure 9:**
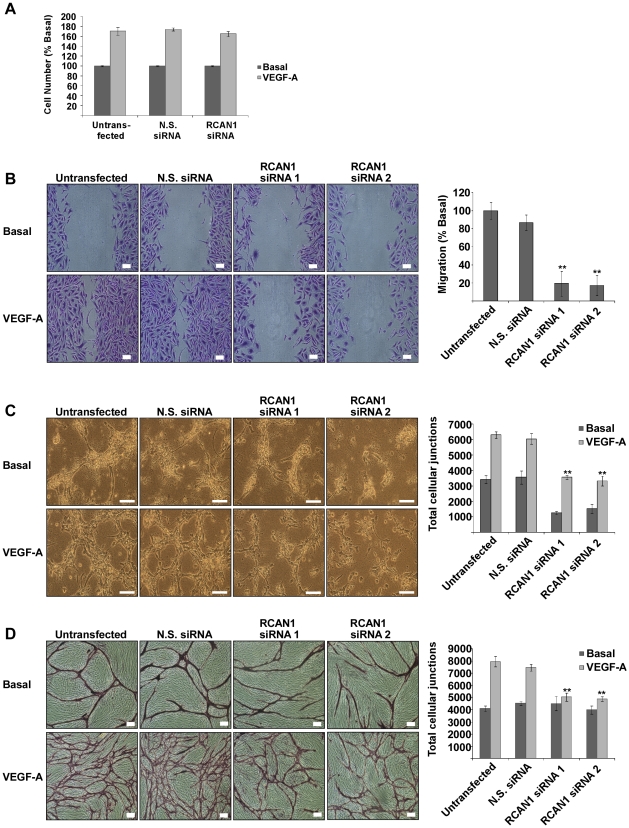
RCAN1.4 is required for migration and tubular morphogenesis, but not proliferation in HDMEC. (*A*) HDMEC were transfected with a mixture of two siRNAs to RCAN1 or non-silencing (N.S.) siRNA and stimulated with VEGF-A (50 ng/ml) for 72 hours. Cellular proliferation was determined by measuring viable cells. Data shows mean ± SD, (n = 3). (*B*) HDMEC were transfected with two individual siRNAs to RCAN1 or N.S. siRNA as indicated. A scratch was introduced into the cell monolayer and the cells then stimulated with VEGF-A (50 ng/ml). Cells were fixed at 18 hours and stained with crystal violet. Bars = 100 µm. The graph shows the extent of migration in response to VEGF-A relative to the untransfected condition. Data shows mean ± SD, (n = 3). **P<0.01, versus untransfected (*C*) Cells were transfected with two individual siRNAs to RCAN1 or N.S. siRNA and plated onto a collagen matrix, and stimulated with VEGF-A (50 ng/ml). Collagen gels were fixed at 20 hours to allow visualisation of tubes. Bars = 100 µm. The graph shows quantification of tubular morphogenesis as measured by the number of cellular junctions. Data shows mean ± SD, (n = 3). **P<0.01, versus untransfected, VEGF treated. (*D*) Cells were cultured upon fibroblast lawns, and transfected with two individual siRNAs to RCAN1 or N.S. siRNA. Cells were stimulated with VEGF-A (50 ng/ml) for 5 days. Cells were fixed and stained for the endothelial specific marker CD31 to allow visualisation of tubes. Bars = 100 µm. The graph shows quantification of tubular morphogenesis as measured by the number of cellular junctions. Data shows mean ± SD (n = 3). **P<0.01, versus untransfected, VEGF treated.

To investigate the role of RCAN1.4 in cellular proliferation in response to VEGF-A stimulation RCAN1.4 expression was knocked down using siRNA and the extent of cellular proliferation over 72 hours was determined. In untransfected cells there was an 80% increase in cell number following VEGF-A stimulation, compared to those cells left in basal conditions. Knockdown of RCAN1.4 in HDMECs did not impact upon cellular proliferation in response to VEGF-A (Fig. 9A).

The role of RCAN1.4 in cellular migration was determined using a scratch wound migration assay, in which RCAN1.4 expression was knocked down, and the ability of the cells to migrate into a scratched wound in response to VEGF-A stimulation was quantified (Fig. 9B). Following knockdown of RCAN1.4 the ability of HDMEC to migrate into the wound was reduced by 80%, indicating a role for RCAN1.4 in endothelial cell migration.

The importance of RCAN1.4 expression in tubular morphogenesis was assessed using two *in vitro* models of angiogenesis. Firstly, the cells were plated within a collagen gel, and stimulated with VEGF-A (Fig. 9C). The ability of cells to form tubes was determined by measuring the total cellular junctions using AngioQuant software [37]. In response to VEGF-A, the number of cellular junctions increased by 85%, when compared with unstimulated cells. However, when cells were transfected with siRNA to RCAN1.4 their ability to undergo tubular morphogenesis was impaired. Under basal conditions the number of cellular junctions was reduced by 70%. Though VEGF-A was able to induce an increase in tubular morphogenesis in cells transfected with RCAN1.4 siRNA, compared to unstimulated cells, it did not exceed the level seen in untransfected basal cells.

To further investigate the importance of RCAN1.4 in tubular morphogenesis a second *in vitro* assay was utilised, in which HDMEC were cultured in combination with human dermal fibroblast cells [21]. As with the collagen assay, the ability of HDMECs to undergo tubular morphogenesis in response to VEGF-A was impaired following knockdown of RCAN1.4 (Fig. 9D). Taken together, the data from the collagen gel assay and co-culture assay suggest that RCAN1.4 is required for efficient tubular morphogenesis.

## Discussion

VEGF-A has previously been shown to upregulate RCAN1.4 expression in endothelial cells, triggering a negative feedback loop which regulates the activity of calcineurin, and results in decreased angiogenesis [10], [17], [38]. It has been previously assumed that VEGF-A is able to regulate RCAN1.4 expression by activation of the classical Ca^2+^/calcineurin pathway leading to activation of the NFAT transcription factor; the potential involvement of other signalling pathways has not been investigated. We now show that in addition to the Ca^2+^/calcineurin pathway, VEGF-A also utilises PKCδ in the regulation of RCAN1.4 expression. In addition, we show that RCAN1.4 is required for efficient tubular morphogenesis in two *in vitro* assays of angiogenesis, including a co-culture model.

RCAN1.4 was found to be upregulated at both the mRNA and the protein level in response to VEGF treatment (Fig. 1B). Although RCAN1.1 has also been reported to be upregulated in HUVECs in response to VEGF [39], we did not see upregulation of RCAN1.1 in HDMECs. Furthermore, basal levels of *RCAN1.1* mRNA were 100-fold lower than basal levels of *RCAN1.4* mRNA, and RCAN1.1 expression was practically undetectable on a western blot. VEGF-A and VEGF-E, but not VEGF-B induced RCAN1.4 expression in HDMECs, indicating that RCAN1.4 upregulation occurs via VEGFR-2 signalling (Fig. 1C). No other growth factor was found to induce RCAN1.4 expression (Fig. 1E and 1F). Furthermore, VEGF-A was the only growth factor found to phosphorylate PLC-γ, and raise intracellular Ca^2+^ (Fig. 1D/2B). Although there is evidence that other growth factors, including FGF-2 and EGF, are able to stimulate PLC-γ activity in other cell lines [40], [41], [42], it would appear that in primary HDMEC the ability of VEGF to stimulate PLC-γ activity is unique amongst growth factors.

In addition to VEGF-A, PMA and A23187 were also found to upregulate the expression of RCAN1.4. Treatment of HUVECs with a combination of VEGF-A and PMA has previously been shown to enhance the upregulation of RCAN1.4 [17]. The ability of PMA to cause RCAN1.4 upregulation has been previously noted in the literature [17], [38], although to date the exact role of PKC in the regulation of RCAN1.4 expression has not been investigated.

Here we show that unlike VEGF-A, stimulation of HDMECs with PMA does not result in the dephosphorylation of NFAT (Fig. 1D). Furthermore, use of the calcineurin inhibitor CsA did not inhibit PMA induced upregulation of RCAN1.4 (Fig. 4A). Finally, we have shown that stimulation with PMA does not induce a rise in intracellular Ca^2+^, which is required for calcineurin activity (Fig. 2B). Taken together, these data point towards a secondary pathway, involving PKC, and independent from the calcineurin pathway, which regulates RCAN1.4.

Profiling of the PKC isoforms in HDMECs detected the expression of just 5 isoforms; the classical α, the novel δ, ε, and η and the atypical ι (Fig. 6A). VEGF-A was found to induce the rapid phosphorylation of PKC α, δ, ε and η, with only PKCδ phosphorylation being sustained, whereas PMA induced sustained activation of these PKC isoforms (Fig. 5). FGF-2 was found to only weakly phosphorylate PKCε and η. This is the first data profiling the temporal phosphorylation of multiple PKC isoforms in response to VEGF-A and FGF-2 in human endothelial cells. Activation of PKCδ has been previously observed following stimulation of endothelial cells with VEGF-A, and has been implicated in Akt activation, and secretion of von Willebrand factor (vWF) from endothelial cells, suggesting that PKCδ may regulate a number of pathways required for endothelial cell function. [43], [44]. Why the phosphorylation of PKCδ is selectively sustained in response to VEGF-A is unknown, but may be related to the activation and interaction of other VEGF-A responsive proteins with PKCδ.

Targeted knockdown of these PKC isoforms using specific siRNAs was performed to further identify which PKCs had a role in RCAN1.4 upregulation (Fig. 6C–D). Following PKCδ knockdown VEGF-A induced RCAN1.4 was reduced at both the mRNA and the protein level by approximately 50%, suggesting that PKCδ is able to regulate RCAN1.4 expression in HDMEC. In contrast, PMA appears to preferentially use PKCε, rather than PKCδ, to regulate RCAN1.4 expression. This is likely to be due to differences in kinetic profiles of phosphorylated PKCs following stimulation by either VEGF-A or PMA in HDMECs. In contrast to VEGF-A, PMA stimulates a sustained phosphorylation of PKCε and η, and it is possible that in this instance PKCε is able to regulate RCAN1.4 expression independently of calcineurin mediated activation of NFAT. PKCδ was also found to be required for VEGF-A induced RCAN1.4 expression in both HUVEC and HAEC confirming that the importance of PKCδ in VEGF-A signalling is not confined to HDMEC (Fig. 6E).

The induction of RCAN1.4 by VEGF-A, but not PMA, was inhibited by the use of the calcineurin inhibitor, CsA. Calcineurin signalling is therefore necessary for RCAN1.4 induction by VEGF-A. However, since knockdown of PKCδ does not result in the complete inhibition of RCAN1.4 induction by VEGF-A, it would appear that signalling via PKCδ may cooperate with the calcineurin pathway to induce RCAN1.4 expression, probably by regulating the activity of a transcription factor distinct from NFAT. A recent study demonstrated that RCAN1.4 could be upregulated by c-Jun, a component of the AP-1 complex [11]. However, it is unlikely that this is the pathway through which PKC is acting as a known inhibitor of AP-1 activation, MEK1 inhibitor U0126 [30], was found not to affect VEGF or PMA stimulated *RCAN1.4* mRNA expression (Fig. 3).

Proliferation is an important process contributing towards angiogenesis. The role of RCAN1.4 in proliferation is still somewhat ambiguous. Overexpression of RCAN1 has been shown to reduce endothelial cell proliferation [10], while knockdown of RCAN1.4 has been shown to increase endothelial cell proliferation [39]. RCAN1.4 is thought to exert its effects upon cellular proliferation via the regulation of calcineurin and NFAT induced gene transcription. In our study we were not able to detect an effect of RCAN1.4 knockdown upon proliferation (Fig. 9A). A similar result has also been reported in HUVECs [16]. A recently published study has shown that knockdown of RCAN1.4 decreases MAPK activation and cellular proliferation in human U87MG glioblastoma cells, via a calcineurin independent mechanism [45]. We do not observe any alterations in MAPK signalling following RCAN1.4 knockdown (Fig. 7A), which is consistent with the lack of effect of RCAN1.4 on cell proliferation.

Directed endothelial cell migration in response to VEGF stimulation represents one of the earliest steps in sprouting angiogenesis [46]. Our data shows that RCAN1.4 is able to regulate migration in HDMEC. RCAN1.4 has previously been shown to have a role in angiogenesis *in vitro*, and *in vivo* [10], [16]. Here we provide data to show that RCAN1.4 expression is required for efficient tubular morphogenesis in a co-culture model (Fig. 9D). Upon stimulation with VEGF-A, HDMEC undergo tubular morphogenesis, which may be quantified using image analysis software. Following knockdown of RCAN1.4 the ability of HDMECs to undergo tubular morphogenesis was impaired. Though the formation of tubular networks can still occur it would appear that the cells are no longer equipped to fully respond to VEGF-A stimulation. Similarly, HDMECs plated upon a collagen gel were less able to form tubular networks in response to VEGF-A following RCAN1.4 knockdown (Fig. 9C). Visual analysis of the tubes upon a collagen gel shows that the tubes that are formed appear to be less organised.

RCAN1.4 is known to have a role in the regulation of tumour angiogenesis. Angiogenesis is a multi-step process requiring the proteolytic digestion of the basement membrane, and co-ordinated migration, proliferation, and finally differentiation of endothelial cells into a lumen-containing vessel supported by pericytes [47]. Here we provide evidence that RCAN1.4 is required for the coordination of specific components of angiogenesis.

**Figure 10:**
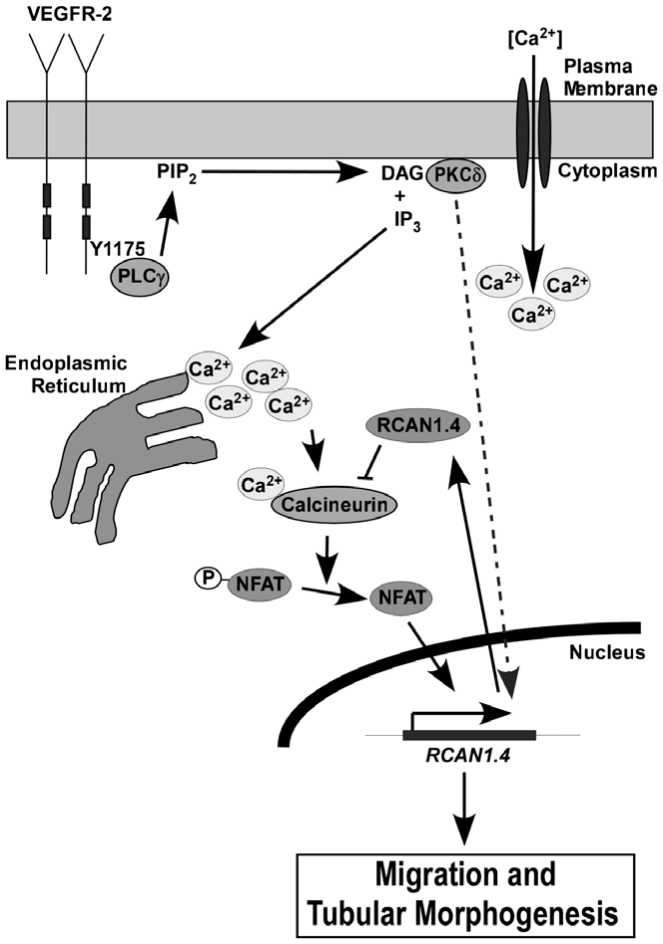
Summary of RCAN1.4 upregulation by VEGF-A in HDMEC. VEGFR-2 activation leads to PLC-γ activation and the subsequent increase in intracellular Ca^2+^ leading to activation of calcineurin and NFAT, which gives rise to increased RCAN1.4 expression. In addition, VEGFR-2 activation results in the sustained phosphorylation of PKCδ, which contributes to the upregulation of RCAN1.4 via an unknown pathway. RCAN1.4 acts to regulate VEGFR-2 mediated cellular migration and tubular morphogenesis.

It has been previously thought that VEGF induces RCAN1.4 via the activation of the Ca^2+^/calcineurin/NFAT pathway. We now provide evidence for a role of PKCδ in the upregulation of RCAN1.4 expression via an independent pathway (Fig. 10). In addition, we show that VEGF appears unique amongst growth factors in its ability to induce an increase in intracellular Ca^2+^ levels, as well as sustained PKCδ phosphorylation, both of which contribute to the upregulation of RCAN1.4 expression in endothelial cells.
